# Forecasting Next-Day Alcohol Craving in Individuals With Problematic Alcohol Use With Multimodal Data and a Tiny Recurrent Neural Network: Prospective Longitudinal Study

**DOI:** 10.2196/101973

**Published:** 2026-11-11

**Authors:** Dongwon Lee, Ji-Yoon Lee, Hyeonmin Lee, Mina Kwon, Suyeon Song, Jeonghyun Yoon, Young-Chul Jung, Woo-Young Ahn, Jung-Seok Choi

**Affiliations:** 1 AI Institute, Seoul National University, Seoul, Republic of Korea; 2 Center for Artificial Intelligence in Healthcare, Seoul National University Bundang Hospital, Seongnam, Gyeonggi-do, Republic of Korea; 3 Department of Psychology, Seoul National University, Seoul, Republic of Korea; 4 Blue Signum, Seoul, Republic of Korea; 5 Institute of Behavioral Science in Medicine, Yonsei University College of Medicine, Seoul, Republic of Korea; 6 Department of Psychiatry, Yonsei University College of Medicine, Seoul, Republic of Korea; 7 Department of Brain and Cognitive Sciences, Seoul National University, Seoul, Republic of Korea; 8 Department of Psychiatry, Samsung Medical Center, Sungkyunkwan University School of Medicine, 81 Irwon-ro, Gangnam-gu, Seoul, 06351, Republic of Korea, 82 2-3410-3581, 82 2-3410-0050

**Keywords:** alcohol craving, artificial intelligence, digital phenotypes, ecological momentary assessment, multimodality, recurrent neural network, smartphone applications

## Abstract

**Background:**

Alcohol craving is associated with drinking behaviors despite negative consequences and relapse following periods of abstinence or treatment. Accurately forecasting alcohol craving is crucial for preventing alcohol consumption and relapse, thereby mitigating alcohol-related problems. Previous studies have assessed alcohol craving using diverse modalities such as psychological assessments, neurophysiological measures including electroencephalography (EEG) or heart rate variability (HRV) at a single time point, and digital phenotypes collected longitudinally via smartphone apps. However, alcohol craving is inherently heterogeneous and multidimensional across individuals and over time, making it difficult to capture using any single modality alone. In contrast, multimodal AI enables the prediction of alcohol craving by integrating complementary behavioral and neurophysiological features.

**Objective:**

This study aimed to develop and evaluate AI models to forecast next-day alcohol craving in individuals with problematic alcohol use with multimodal datasets.

**Methods:**

We analyzed multimodal datasets comprising smartphone-based digital phenotypes collected over 28 days, along with baseline EEG, HRV, and psychological measures in a prospective longitudinal study of 60 participants with problematic alcohol use. A tiny multimodal recurrent neural network (RNN) was implemented, in which representations of static baseline features were encoded into a latent representation and used to initialize the hidden state of the RNN. Model performance was evaluated using accuracy, weighted *F*_1_-score, macro *F*_1_-score, and mean absolute error (MAE).

**Results:**

The multimodal model consistently outperformed the unimodal model based on digital phenotypes alone, achieving higher accuracy (0.56 vs 0.45), weighted *F*_1_-score (0.51 vs 0.38), and macro *F*_1_-score (0.37 vs 0.24), along with lower MAE (0.68 vs 0.90). At the individual level, 72% (43/60) of participants showed improved prediction accuracy with the multimodal model. These improvements were statistically significant (Wilcoxon signed-rank test, *P*<.001; paired *t* test, *P*<.001).

**Conclusions:**

Integrating digital phenotypes with neurophysiological and psychological features enhances the prediction of short-term alcohol craving dynamics. This approach may enable more precise and timely personalized interventions for individuals at risk of relapse.

## Introduction

According to the 2024 World Health Organization Alcohol Fact Sheet, alcohol consumption was responsible for approximately 2.6 million deaths worldwide in 2019 [1]. Alcohol consumption is linked to an increased risk of mental health conditions such as depression, anxiety, and alcohol use disorders (AUDs), as well as liver disease, heart disease, and cancer [1]. AUD is a chronic relapsing disorder that affects physical, psychological, and social functioning, highlighting the need for sustained treatment and ongoing clinical care [2]. The disorder is a leading contributor to illness and death but is frequently improperly diagnosed or treated in clinical settings, and as a result, the burden of disease remains high [2]. Problematic alcohol use spans a wide spectrum ranging from nonclinical binge drinking to AUD [3]. Therefore, early identification and intervention are important before drinking behaviors progress to more severe alcohol-related problems [4].

Alcohol craving is a critical factor in problematic alcohol use, contributing to persistent drinking behavior as well as relapse and the maintenance of alcohol dependence [5,6]. Craving has traditionally been regarded as a primary driver of alcohol use, with cognitive processing models suggesting that alcohol consumption often occurs through automatized behaviors, whereas craving reflects a conscious cognitive response to the disruption or inhibition of drinking processes [7]. Sociodemographic disparities in alcohol craving and AUD outcomes have also been reported in previous studies [8]. Assessing craving may help clarify its association with alcohol relapse in predicting drinking during treatment [9]. Alcohol craving is a time-dependent event that varies across situations and contexts and requires repeated assessments in natural environments [10]. It was shown to predict subsequent alcohol use, and higher craving levels were associated with an increased likelihood of drinking and heavy drinking in the following week [11]. This predictive association appears to be strongest over short time frames and identifies craving as a key indicator of forthcoming drinking risk [11]. However, many previous studies have assessed craving at a single modality and time point using cross-sectional approaches, which may fail to capture the dynamic fluctuations of craving in daily life [12]. Therefore, optimal measurement of alcohol craving requires assessing it at least daily, particularly in high-risk situations for drinking or when craving significantly impacts functioning [12].

Given that alcohol craving fluctuates dynamically across time and contexts, continuous real-time monitoring through digital phenotyping may provide a promising approach for capturing these temporal variations in naturalistic settings. Advances in digital technologies and data analytics have created unparalleled opportunities to assess and modify health behavior and thus accelerate the ability of science to understand and contribute to improved health behavior and health outcomes [13]. Digital phenotyping measures behavior from smartphone sensors, keyboard interaction, and various features of voice and speech. It can capture diverse variables spanning psychomotor activity, mood state, geolocation, and movement in real time, with a potential to assess features related to specific addictive behaviors, such as urges, cravings, lifestyle factors, and high-risk environmental situations [14,15]. In a recent study, alcohol craving exhibited a systematic rhythm over the course of a 24-hour period, and alcohol use frequency was associated with the shape of these rhythms [16]. Findings suggest that consideration of daily rhythms in alcohol craving may further enhance the understanding of mechanisms driving alcohol use [16]. This approach would be useful for assessing craving as a time-dependent event, and further research is needed to validate its utility.

While digital phenotyping provides valuable insights into momentary behavioral states in everyday life, alcohol craving is also influenced by stable individual characteristics related to addiction vulnerability. Neurophysiological measures such as electroencephalography (EEG) and heart rate variability (HRV) have been associated with reward processing, emotional regulation, and autonomic functioning in substance use disorders. Resting-state EEG findings indicate that subjective craving is associated with frontal beta activity, reflecting an electrophysiological correlate of alcohol craving induced by personalized cues [17]. A previous functional magnetic resonance imaging–EEG study showed that alcohol craving was associated with abnormal activity in key brain regions, including the dorsal anterior cingulate cortex, nucleus accumbens, posterior cingulate cortex, pregenual anterior cingulate cortex, amygdala, and parahippocampus, and was also linked to increased beta-band activity in resting-state EEG [18]. Another HRV study showed that high-frequency HRV is an important predictor of alcohol craving, supporting its role as a potential biomarker of craving in individuals with alcohol dependence and its involvement in self-regulatory processes [19]. These trait-level biomarkers may reflect underlying neurobiological susceptibility to craving and relapse and should therefore be considered alongside dynamic behavioral data in alcohol craving prediction.

In this context, psychiatric features are heterogeneous and multidimensional, and vary across individuals, and therefore may not be adequately captured by unimodal approaches [20]. Digital phenotyping captures temporal fluctuations in real-world behavior but overlooks underlying neurobiological vulnerability, whereas neurophysiological studies identify trait-level markers but may lack sensitivity to time-varying dynamics. To address these limitations, a previous review has highlighted the integration of neuroimaging and digital phenotyping, demonstrating its potential to link behavioral dynamics with underlying neural mechanisms [21]. However, this approach remains in its early stages, with key challenges including temporal misalignment between modalities and the limited use of multivariate analyses and machine learning (ML) approaches [21]. Recent advances in AI and ML provide powerful approaches for modeling complex interactions across multimodal datasets [22]. ML approaches are particularly well-suited to identifying temporal misalignment and modeling complex, time-varying relationships across modalities. This integration may provide a more comprehensive understanding of alcohol craving.

Taken together, this study aims to predict next-day alcohol craving in individuals with problematic alcohol use using multimodal datasets. To account for temporal discrepancies across modalities, we used a tiny recurrent neural network (RNN) approach that explicitly models time-dependent relationships. We compared AI algorithms trained on only longitudinal digital phenotypes collected via a smartphone app over 28 days with those on a fully integrated dataset using digital phenotypes, EEG, HRV, and psychological features. We hypothesized that an AI model with a fully integrated multimodal dataset would outperform those using only longitudinal digital phenotypes.

## Methods

### Participants

The study participants were adults aged 19 to 59 years. The upper age limit was set to minimize potential confounding effects of age-related neurophysiological and structural brain changes [23-26]. The sample size was informed by previous EEG and ML studies in alcohol-related populations with comparable sample sizes [27-29]. A total of 70 participants who self-reported problematic drinking behaviors were recruited. Participants were recruited between July 2023 and July 2024 through an online advertisement posted on AllLiveC, a platform specializing in clinical trial and research participant recruitment, targeting individuals with self-reported problematic drinking behaviors. Participants were not receiving formal treatment for AUD during the study period. Exclusion criteria included inability to understand the consent form or provide informed consent, pregnancy or plans for pregnancy, participation in another clinical trial, use of more than 2 phones, and claustrophobia or contraindications for magnetic resonance imaging scanning. Two participants withdrew early from the study, and 7 participants were excluded after study initiation due to age, failure to enter alcohol information in the mobile app, or missing visit 1. Additionally, 1 participant was excluded due to excessive discontinuities that prevented the construction of valid sequential windows. As a result, the final sample consisted of 60 participants in the final analysis (Figure 1).

**Figure 1.**
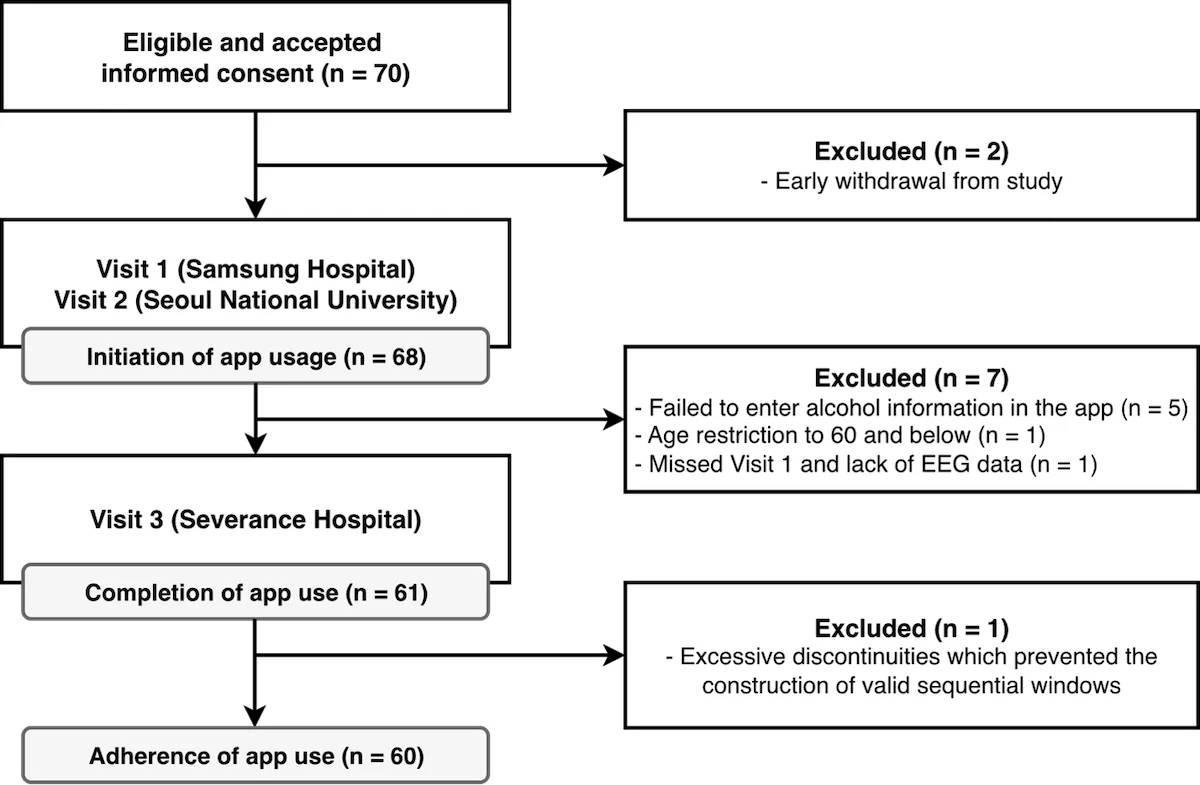
Participant flow diagram.

### Ethical Considerations

The study protocol was approved by the institutional review board of Severance Hospital, Yonsei University Health System (4-2023-1590) and Samsung Medical Center (2023-02-115), Seoul, South Korea. All participants provided informed written consent before enrollment. To protect participant privacy and confidentiality, participant personal information was managed in a pseudonymized form throughout the study. Participants received compensation according to their level of app-use participation, with compensation averaging approximately KRW 200,000 (US $149) per participant.

### Assessments by Visit

At the baseline visit (visit 1), participants’ demographic information was collected, and assessments for HRV, EEG, and psychological and cognitive function were conducted. During this visit at Samsung Medical Center, Seoul, participants also completed interviews with the Mini-International Neuropsychiatric Interview to rule out comorbid psychiatric diagnoses and were evaluated for AUD based on *Diagnostic and Statistical Manual of Mental Disorders, Fifth Edition* [30] criteria. Cognitive function was assessed using the Korean Wechsler Adult Intelligence Scale-IV and several psychometric assessments. Additionally, EEG and HRV tests were performed.

On visit 2, the Harujjan application was installed on participants’ mobile devices, and they received detailed, step-by-step instructions on its usage. Participants were guided on how to log their daily entries, including mood, sleep quality, and alcohol intake, and set reminders to ensure consistent usage throughout the study. Ecological momentary assessment (EMA) assessments were administered once daily via a mobile app and were completed at the end of each day at a participant-selected time. Automated push notifications were provided at the selected time, and each survey took approximately 5 minutes to complete. The daily EMA survey comprised 17 items across 4 domains: mood (overall mood and interest/pleasure), diet (meal consumption and meal quantity), alcohol-related behaviors (alcohol craving, alcohol consumption, drinking time, beverage type, amount consumed, drinking companions, drinking duration, drinking location, drinking motives, and blackout experience), and sleep (sleep duration, sleep satisfaction, and sleep-related daytime interference). Some items were conditionally presented based on participants’ responses. If participants consumed additional food or alcohol after completing the survey, they could submit additional entries until the following morning. Surveys were completed daily regardless of whether craving was experienced; no event-contingent assessments were used.

At the final visit (visit 3), after 4-week mobile app usage, participants completed an HRV test and a psychometric assessment.

### Datasets

#### Outcome and Predictors

The primary outcome was next-day alcohol craving, assessed using a single-item EMA (“How much did you want to drink alcohol today?”), rated on a 5-point Likert scale ranging from 1 (not at all) to 5 (very much). For modeling, this outcome was treated as a 5-class classification target, with the craving level reported on the following day serving as the prediction target.

Predictors comprised longitudinal EMA-based time-series features, including mood, dietary, sleep, and alcohol consumption measures, and static baseline features, including cognitive, alcohol-use, psychological, EEG, and HRV measures.

#### Psychological Features

Psychological features included Drinking Motives Questionnaire (DMQ) [31,32] scores for social, coping, enhancement, and conformity; Alcohol Use Disorders Identification Test (AUDIT) [33,34], Beck Depression Inventory-II (BDI-II) [35,36], Beck Anxiety Inventory (BAI) [37,38]; raw and transformed Barratt Impulsiveness Scale (BIS) [39,40] total score and subscales for attentional, motor, and nonplanning; Pittsburgh Sleep Quality Index (PSQI) [41,42] total score and subscales for subjective sleep quality, sleep latency, sleep duration, sleep efficiency, sleep disturbance, use of sleep medication, and daytime dysfunction; and Stages of Change Readiness and Treatment Eagerness Scale (SOCRATES) [43,44] subscales for ambivalence, recognition, and taking steps. All of those scales were validated for Korean use. Intelligence quotient was assessed by the Korean Wechsler Adult Intelligence Scale-IV [45].

#### EEG

Resting-state EEG was recorded in a sound-shielded room for 10 minutes: 5 minutes with eyes closed and 5 minutes with eyes open, using a 64-channel Quik-Cap (Compumedics Neuroscan) following the modified International 10-20 system. All EEG recordings were obtained using SynAmps 2 (Compumedics) and the Neuroscan system (Scan 4.5; Compumedics). The electrode impedance was maintained below 5 kΩ. EEG data were processed using Python (version 3.12.2), MNE-python (version 1.8.0) [46], and autorejection [47]. Artifact-free 2-second epochs from the eyes-closed condition were selected for analysis. The relative power was computed for 5 frequency bands: delta (1‐4 Hz), theta (4‐8 Hz), alpha (8‐12 Hz), beta (12‐25 Hz), and gamma (30‐40 Hz).

#### HRV

HRV data were obtained during a 5-minute resting-state recording. Participants remained seated while electrocardiogram electrodes were placed on both wrists and the left ankle. Electrocardiogram signals were recorded using a ProComp Infiniti system (SA7500 Computerized Biofeedback System; Thought Technology Ltd) equipped with an electrocardiogram-Flex/Pro sensor, with a sampling rate of 256 Hz and a band-pass filter of 0.26‐30 Hz. Participants were asked to abstain from caffeine and nicotine for at least 2 hours before the recording. HRV features included the following: average beats per minute, standard deviation of normal-to-normal intervals, physical stress index, total power, very low-frequency power, low-frequency power, high-frequency power, normalized low-frequency power, normalized high-frequency power, low-frequency to high-frequency ratio, root mean square of successive differences, approximate entropy, short-range detrended fluctuation analysis, total short-range detrended fluctuation analysis, natural logarithm of total power, natural logarithm of very low-frequency power, natural logarithm of low-frequency power, and natural logarithm of high-frequency power.

### Data Preprocessing

#### Handling Missing Data

Participants were instructed to complete EMA entries for 28 consecutive days. However, adherence varied substantially across individuals. While some participants provided entries beyond the 28-day period, others reported fewer observations, and many exhibited irregular reporting patterns with intermittent day gaps between consecutive days. Among the final analytic sample of 60 participants, 29 provided EMA responses for exactly 28 days, while the number of EMA response days ranged from 21 to 30 across participants (Figure S3 in Multimedia Appendix 1). Additionally, 4 participants exhibited nonconsecutive reporting patterns with day gaps. Given the sequential nature of our modeling framework, we did not impute missing days to artificially construct continuous time series, as such imputation may introduce unrealistic temporal dependencies and bias the learning process [48]. Instead, we adopted a sliding window framework and excluded invalid sequences based on temporal consistency. Specifically, we removed (1) windows containing gaps greater than one day within the input sequence and (2) windows where the temporal gap between the final input observation and the prediction target exceeded 2 days. This ensured that all training samples preserved meaningful temporal continuity and temporally consistent short-term behavioral patterns. For missing values within individual features, we applied domain-informed imputation strategies tailored to each variable type.

#### Feature Selection

After data preprocessing, the dataset consisted of 394 baseline features with 60 samples and 45 time-series features from the app-based EMA data. Given the high-dimensional feature space relative to the limited sample size (ie, *P*>>N), the model was highly susceptible to overfitting, which can degrade generalization performance on unseen datasets [49]. Therefore, feature selection was a critical step to reduce the dimensionality and improve model robustness [50]. Among various methods, random forest–based feature selection is widely used for high-dimensional, low-sample-size settings [51]. Accordingly, random forest–based feature selection was applied separately to the EMA (time-series) data and baseline (static) data.

For the EMA data, feature importance was computed using a random forest classifier trained exclusively on the training portion of the dataset, excluding the final 8 days per participant used for validation and testing to prevent data leakage. Features with importance values above the mean importance threshold were retained. For the baseline data, random forest regression was applied using the participant-level average alcohol craving as the target variable. The average craving was computed using only the training data (excluding the final 8 days) to prevent information leakage. Based on random forest feature importance, 42 baseline features were retained. After feature selection, 11 features remained for the EMA data and 42 features for the baseline data, substantially reducing the feature space while preserving the most informative predictors. One additional feature derived from EEG relative power (epoch count) was manually excluded, as it was not considered a meaningful physiological predictor but rather reflected data preprocessing characteristics.

Consequently, a total of 41 baseline features were retained for subsequent analyses. This separate feature selection strategy was adopted to account for the fundamentally different statistical properties of time-series and static data. Selected EMA features included mood (Patient Health Questionnaire) items, dietary patterns (meal frequency), sleep quality, and alcohol consumption variables. Selected baseline features included cognitive measures (eg, intelligence quotient and working memory index), alcohol-use measures, psychological scales (eg, DMQ, BIS, and SOCRATES), EEG band power across multiple regions (delta, theta, beta, and gamma), and HRV-derived metrics. A full list of the selected features of the baseline and the EMA data is provided in Tables S1 and S2 in Multimedia Appendix 1.

#### Data Splitting Strategy (Sliding Window Approach)

Our objective was to predict the next-day alcohol craving using prior observations. Forecasting refers to the prediction of future outcomes based on past and present information [52,53]. In time-series forecasting, a sequence of past observations, often referred to as a sliding window, is used to predict future targets [54]. When evaluating the performance of time-series models, it is critical to ensure that no future information is used during training, and that only data available at the time of prediction are used for parameter estimation [53]. To preserve the temporal structure of the data and prevent information leakage, a participant-wise time-series split was applied. For each participant, the last 5 days were used as the test set, and the preceding 3 days were used as the validation set. The remaining observations were used for training. This approach ensures that the model is evaluated on future data that were not available during training, thereby reflecting a realistic forecasting scenario while preserving temporal ordering and preventing information leakage from future observations. The choice of lookback-window length involves a trade-off between retaining sufficient temporal context and avoiding irrelevant or noisy historical information [55]. We therefore examined the robustness of different window lengths, with detailed sensitivity analyses for 2-, 4-, 6-, and 8-day windows provided in Table S4 in Multimedia Appendix 1. A sliding window approach with a window size of 4 was used for the primary analyses, providing a short-term temporal context for next-day forecasting.

### Feature Scaling

Feature scaling is an essential preprocessing step in ML that ensures variables are on a comparable scale, thereby improving model stability and performance [56]. We applied standardization using the standard scaler, a widely used normalization technique [57]. Standardization transforms each feature to have zero mean and unit variance, computed as:


(1) $$z=\frac{X-\mu }{\sigma }$$

where *z* is the transformed value, *X* is the original feature value, *μ* is the mean, and *σ* is the SD [58]. To prevent data leakage, the scaler was fitted exclusively on the training data. The same transformation parameters were then applied to the validation and test sets.

### Modeling

#### RNNs

Traditional clinical approaches often integrate static and time-series data by extracting statistical features from the temporal data, which are then combined with static features. However, such approaches remove the temporal structure of the data and require manual engineering of temporal features, which can be labor-intensive and may result in the loss of temporal information [13,59]. In contrast, RNNs are designed to model sequential data and can capture temporal dependencies directly from raw time-series inputs, enabling more effective use of temporal information [60]. However, clinical datasets typically consist of both static (eg, EEG, HRV, and psychological assessments) and dynamic (EMA time-series) features. Due to their nonsequential nature, static features cannot be incorporated into standard RNN architectures [61,62].

To address this limitation, we adopted a tiny (lightweight) RNN framework that facilitates the integration of multimodal data while maintaining model simplicity. The use of a compact architecture is particularly important given the limited sample size, as it reduces the risk of overfitting while preserving the ability to capture meaningful temporal patterns. Furthermore, we hypothesize that incorporating highly informative multimodal baseline features such as EEG, HRV, and psychological assessments [18,19] allows the model to leverage complementary information that is not directly observable from EMA data alone, thereby improving model performance.

#### Training the Unimodal Dataset

To establish a baseline for evaluating the contribution of multimodal integration, the model was first trained using only the EMA time-series data. A tiny vanilla RNN framework with a single hidden layer of 16 units was adopted to model temporal dependencies while minimizing the risk of overfitting given the limited sample size. The hidden state of the vanilla RNN is updated at each time step according to:


(2) $$h_{t}=tanh(W_{xh}X_{t}+W_{hh}h_{t-1}+b)$$

where $X_{t}\in R^{n}$ is the input at time step *t*, $h_{t}\in R^{m}$ is the hidden state, and *b* is the bias term. The hidden state serves as a dynamic representation of temporal information and is initialized as a zero vector [63]. Given that our target variable is encoded on a Likert scale, the task was formulated as a 5-class classification problem. Therefore, a softmax layer was applied to obtain class probabilities:


(3) $$p_{i}=\frac{exp\;(z_{i})}{\Sigma_{i}\;exp\;(z_{i})}$$

and the predicted class was defined as:


(4) $$y_{t}=arg\;\underset{i}{max\;}p_{i}$$

where $z_{i}$ denotes the output of the final linear layer and $p_{i}$ is the probability of class *i*. The predicted values for the next-day alcohol craving correspond to the class with the highest probability.

#### Training the Multimodal Dataset

To effectively integrate static and time-series data, we adopted a tiny multimodal RNN framework that initializes the hidden state of the RNN using representations learned from static baseline features. This approach enables the model to incorporate subject-specific contextual information into the temporal dynamics of the sequence model, allowing it to capture temporal dependencies while preserving informative baseline characteristics (Figure 2).

**Figure 2.**
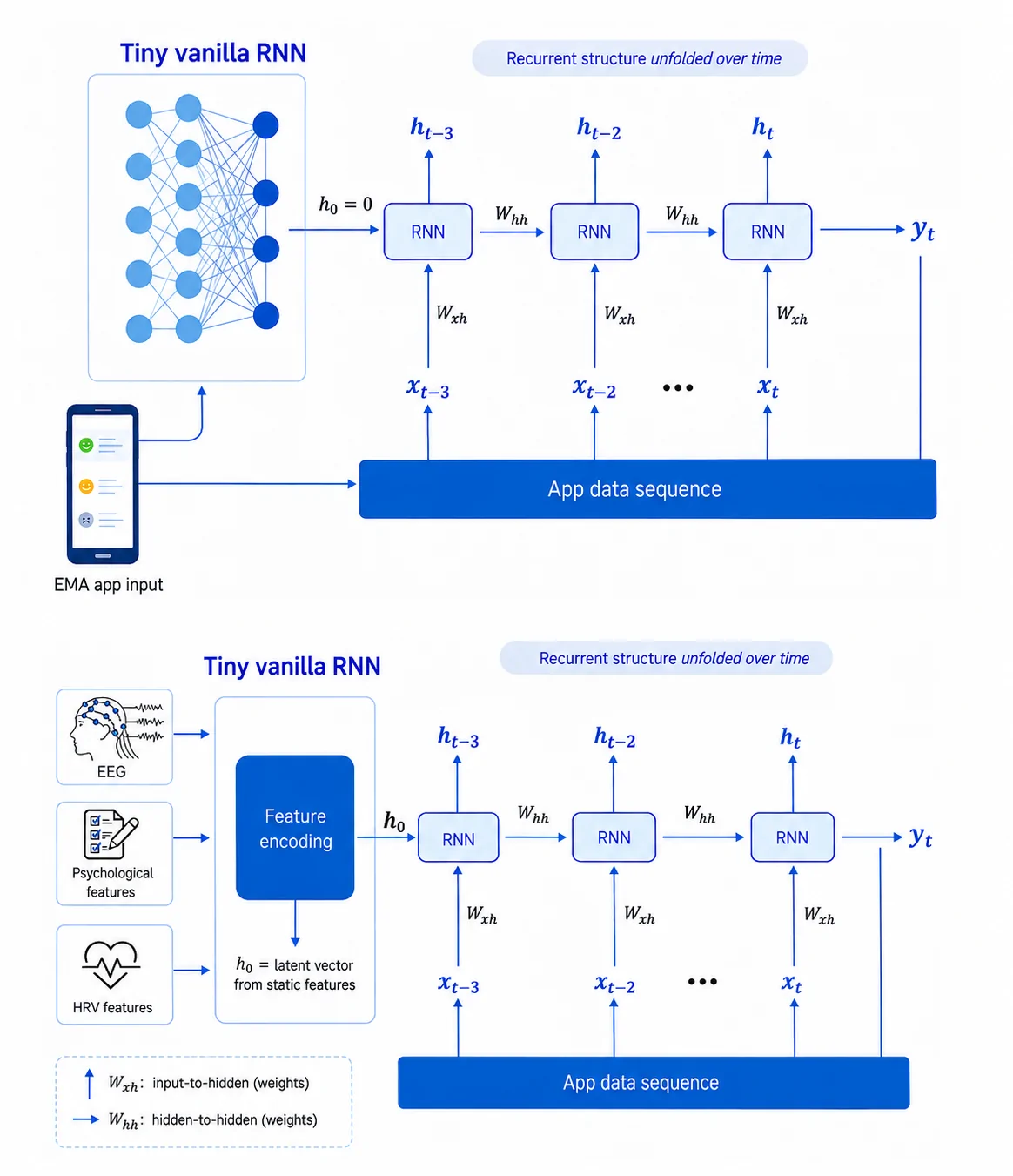
Model architectures. Top: Unimodal model — a vanilla recurrent neural network (RNN) processes ecological momentary assessment (EMA) time-series with the hidden state initialized as a zero vector. Bottom: Multimodal model — static baseline features (electroencephalography [EEG], heart rate variability [HRV], and psychological measures) are encoded into a latent vector via a feedforward network, which initializes the RNN hidden state, allowing individual-specific baseline characteristics to directly condition temporal predictions.

Specifically, static baseline features (eg, EEG, HRV, and psychological assessments) are first transformed into a latent representation using a single-layer feedforward neural network.

This network consists of one hidden layer with a rectified linear unit activation function [64] and a dropout rate of 20% to mitigate overfitting. The resulting representation is then used to initialize the hidden state of the RNN. Subsequently, EMA time-series data are processed sequentially by the RNN, similar to the unimodal setting.

The key difference is that, instead of initializing the hidden state with a zero vector, the multimodal model initializes it with the learned representation of the static baseline features. This allows the model to condition temporal predictions on individual-specific baseline characteristics. The use of a compact architecture is particularly important given the limited sample size (n=60), as it reduces model complexity while maintaining the ability to capture both temporal dependencies and cross-modal interactions.

A key aspect of our approach is the integration of static baseline features as initial hidden states in the recurrent model, allowing subject-specific contextual information to directly influence temporal predictions.

#### Model Training

All models were implemented using the open-source deep learning library PyTorch [65]. The training data were processed in minibatches of size 32 with shuffling enabled, while validation and test data were evaluated using larger batch sizes of 256 without shuffling. To ensure robustness and reduce sensitivity to random initialization, each model was trained 100 times with different random seed values (ranging from 2026 to 2125). Performance metrics were summarized across the 100 runs using the mean, SD, and 95% CI of the mean. The model parameters were optimized using the Adam optimizer [66] with a learning rate of 0.001. The categorical cross-entropy loss was used for training. For both unimodal and multimodal models, training was conducted for a maximum of 70 epochs. Early stopping was applied based on validation loss, where the model parameters corresponding to the lowest validation loss were selected for final evaluation to prevent overfitting.

#### Evaluation Setting

To ensure a realistic forecasting setting, model evaluation was conducted using only information available prior to the prediction time point, preventing the use of future data for parameter estimation [53]. Accordingly, the dataset was split along the temporal dimension for each participant, rather than participant-level splitting. The last 5 days were used as the test set, and the preceding 3 days were used as the validation set for hyperparameter tuning, with the remaining observations used for training.

Model performance was evaluated using multiple metrics to account for class imbalance and the ordinal nature of the target variable. Accuracy is the ratio of the number of correctly classified samples over the total number of samples [67]. Accuracy was reported as an overall performance measure; however, when evaluating imbalanced datasets, accuracy may be misleading, as it considers only the total number of correct predictions without accounting for the distribution of class labels [68]. The imbalanced distribution of our craving labels is shown in Figure S3 in Multimedia Appendix 1.

Therefore, aggregated metrics such as *F*_1_-score were also considered to address the issue of class imbalance [69]. *F*_1_-score is the weighted harmonic mean of precision and recall [70]. While weighted *F*_1_-score is used when there are imbalanced classes, it may be biased toward the majority class [71,72]. On the other hand, macro *F*_1_-score treats all classes equally by assigning the same weight to each class, ensuring that minority classes also contribute to the evaluation and that the model is penalized if it performs poorly on them [73]. Therefore, both metrics were reported.

In addition, since the target variable is ordinal, prediction errors of smaller magnitude are more acceptable than larger deviations. In imbalanced ordinal classification settings, metrics that account for the magnitude of prediction errors, such as mean absolute error (MAE), have been shown to provide informative evaluation [74]. Thus, model performance was evaluated using accuracy, weighted *F*_1_-score, macro *F*_1_-score, and MAE.

To explore participant-level factors potentially associated with prediction performance, post hoc Spearman rank correlations were calculated between participant-wise mean test accuracy and EMA-related characteristics, including EMA response days, within-participant craving SD, craving entropy, the number of unique craving levels, and the number of usable sequential samples. Within-participant craving SD, craving entropy, and the number of unique craving levels were calculated across each participant’s observed EMA period. Correlations with the difference between multimodal and unimodal accuracy (Δaccuracy) were also examined.

## Results

### Demographic Features

The demographic characteristics of the participants are presented in Table 1. The average age of the participants was 34.17 (SD 8.62) years, with 25 males and 35 females included.

**Table 1.** Demographic features of the participants.

Demographic	Value
Age (years), mean (SD)	34.17 (8.62)
Sex	
Male	25 (41.7)
Female	35 (58.3)
IQ, mean (SD)	110.83 (14.12)
Education (years), mean (SD)	15.65 (3.11)
Marriage, n (%)	
Yes	19 (31.7)
No	41 (68.3)
Smoking, n (%)	
Yes	14 (23.3)
No	46 (76.7)
AUDIT^a^ score, mean (SD)	18.15 (6.67)

a AUDIT: Alcohol Use Disorders Identification Test.

### EMA Completion and Craving Characteristics

Among the 60 participants included in the final analytic sample, participants provided EMA responses for a mean of 27.88 (SD 1.35; median 28, IQR 28‐29; range 21‐30) days. A total of 29 (48.3%) participants provided responses for exactly 28 days, while 47 (78.3%) participants provided responses for at least 28 days. Some participants continued to submit EMA entries beyond the planned 28-day period, with 18 (30%) participants providing more than 28 days of data.

Before construction of the next-day prediction samples, a total of 1673 alcohol craving reports were submitted. The mean EMA-reported alcohol craving score was 2.21 (SD 1.13), with a median of 2 (IQR 1‐3) and a range of 1‐5.

### Model Performance

The unimodal and multimodal models were compared across the aforementioned evaluation metrics, including accuracy, weighted *F*_1_-score, macro *F*_1_-score, and MAE. The results of the 5-class classification are reported as the mean, SD, and 95% CI of the mean across 100 runs with different random seeds (Table 2).

**Table 2.** Model performance comparison of unimodal and multimodal models. Values are presented as mean (SD) across 100 runs.

	Train, mean (SD)	Validation, mean (SD)	Test, mean (SD; 95% CI)
Unimodal			
Accuracy	0.46 (0.01)	0.46 (0.02)	0.45 (0.01; 0.443‐0.447)
Weighted *F*_1_-score	0.38 (0.02)	0.39 (0.02)	0.38 (0.01; 0.372‐0.378)
Macro *F*_1_-score	0.26 (0.04)	0.23 (0.02)	0.24 (0.04; 0.235‐0.249)
MAE^a^	0.90 (0.04)	0.85 (0.04)	0.90 (0.03; 0.894‐0.904)
Multimodal			
Accuracy	0.59 (0.02)	0.53 (0.03)	0.56 (0.02; 0.552‐0.560)
Weighted *F*_1_-score	0.53 (0.04)	0.49 (0.03)	0.51 (0.03; 0.504‐0.517)
Macro *F*_1_-score	0.43 (0.06)	0.32 (0.05)	0.37 (0.05; 0.364‐0.381)
MAE	0.62 (0.04)	0.72 (0.04)	0.68 (0.04; 0.672‐0.686)

a MAE: mean absolute error.

Overall, the multimodal model consistently outperformed the unimodal model across all evaluation metrics. In terms of accuracy, the multimodal model achieved 0.56 (SD 0.02; 95% CI 0.552-0.560) on the test set, compared to 0.45 (SD 0.01; 95% CI 0.443-0.447) for the unimodal dataset. Considering that a random classifier would achieve an accuracy of approximately 20% in a 5-class classification setting, and a majority-class classifier would yield approximately 37%, both models demonstrate meaningful predictive performance, with the multimodal model providing substantial improvement.

Given the imbalanced class distribution, *F*_1_-scores were also evaluated. The unimodal model achieved a weighted *F*_1_-score of 0.38 (SD 0.01) and a macro *F*_1_-score of 0.24 (SD 0.04) on the test set, whereas the multimodal model improved these scores to 0.51 (SD 0.03) and 0.37 (SD 0.05), respectively. The improvement in macro *F*_1_-scores suggests that the multimodal model better captures minority class patterns, leading to more balanced performance across classes. Furthermore, MAE was used to account for the magnitude of prediction errors given the ordinal nature of the prediction task. The unimodal model yielded an MAE of 0.90 (SD 0.03), whereas the multimodal model reduced this to 0.68 (SD 0.04). This indicates that the multimodal model not only improves the classification accuracy but also reduces the magnitude of prediction errors, producing predictions that are closer to the true ordinal labels. These results demonstrate that incorporating static baseline features into the temporal modeling framework provides complementary information that enhances both classification performance and error sensitivity.

Sample-level confusion matrices based on the modal prediction across the 100 runs for the unimodal and multimodal models, including raw-count and row-normalized representations, are provided in Figure S4 in Multimedia Appendix 1. The multimodal model showed improved class-specific prediction across several craving levels, including some less frequent classes. Notably, correct classification of craving level 5 increased from 0% in the unimodal model to 33% in the multimodal model, although performance for craving level 4 remained limited.

### Accuracy Comparison at the Individual Level

To further examine whether the multimodal model consistently improves performance at the individual level, we compared participant-wise accuracy between the unimodal and multimodal models. For each participant, the difference in accuracy between the multimodal and unimodal models was computed. The multimodal model consistently outperformed the unimodal model at the individual level, with 72% (43/60) of participants showing higher prediction accuracy with the multimodal model, indicating that the performance improvement was consistent across the majority of individuals. Participant-wise accuracy was significantly higher for the multimodal model than for the unimodal model. This improvement was confirmed by both the Wilcoxon signed-rank test (W=176; *P*<.001) and the paired *t* test (*t*_59_=5.00; *P*<.001), treating the accuracy values as paired observations for each participant. The use of both nonparametric and parametric tests was motivated by uncertainty regarding the normality of the distribution. As illustrated in Figure 3, these findings demonstrate that the multimodal approach improves predictive performance not only at the aggregate level but also consistently across individuals.

**Figure 3.**
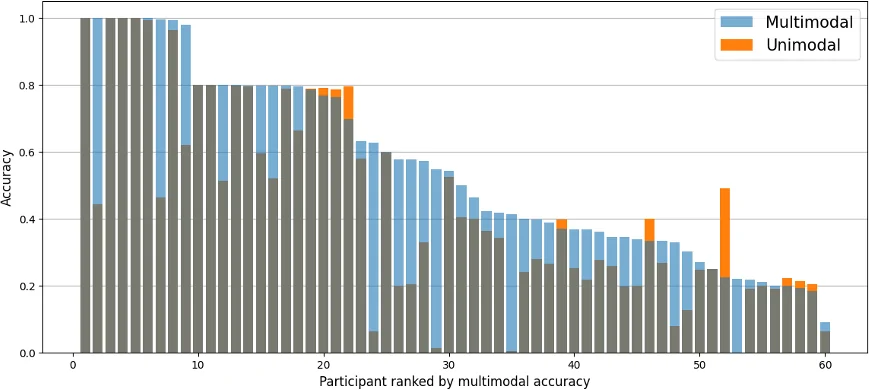
Participant-wise prediction accuracy for the unimodal (orange) and multimodal (blue) models across all n=60 participants. Blue bars indicate the multimodal model, orange bars indicate the unimodal model, and gray bars represent the overlapping region where the two bars coincide. The multimodal model achieves higher accuracy in 72% (43/60) of participants, with statistically significant improvement confirmed by both the Wilcoxon signed-rank test and paired *t* test (both *P*<.001).

To explore potential factors associated with participant-level prediction performance, we conducted post hoc Spearman correlation analyses. EMA response days were not significantly associated with accuracy in either the unimodal (ρ=0.035; *P*=.79) or multimodal (ρ=0.085; *P*=.52). In contrast, greater within-participant craving variability was associated with lower accuracy in both models (unimodal: ρ=−0.549; *P*<.001; multimodal: ρ=−0.671; *P*<.001). Craving entropy and the number of unique craving levels were likewise negatively associated with accuracy in both models, whereas the number of usable sequential samples was not significantly associated with accuracy. None of the examined participant characteristics was significantly associated with Δaccuracy. Detailed results are provided in Table S3 in Multimedia Appendix 1.

### Modality Ablation

To examine the contribution of each baseline modality, we conducted a modality ablation analysis in which each baseline modality was used individually to initialize the RNN hidden state and compared with the unimodal zero-initialization model and the full multimodal model (Table 3). The modality ablation results provide interpretable insight into which signals drive performance gains. HRV features yield the strongest single-modality improvement, consistent with the established role of autonomic regulation in craving and relapse [19]. EEG features also contribute substantially, while psychological features alone produce smaller gains—possibly due to overlap with EMA-collected mood and behavioral data. These findings suggest that clinicians prioritizing resource-constrained deployment might first consider HRV monitoring as a complement to EMA, given its relative ease of collection compared to full EEG acquisition.

**Table 3.** Modality ablation performance on the test set. Values represent means across 100 runs.

Model	Accuracy, mean (SD)	Weighted *F*_1_-score, mean (SD)	Macro *F*_1_-score, mean (SD)	MAE^a^, mean (SD)
Unimodal (zero initialization)	0.45 (0.01)	0.38 (0.01)	0.24 (0.04)	0.90 (0.03)
+ EEG^b^ only	0.52 (0.03)	0.46 (0.04)	0.33 (0.05)	0.76 (0.06)
+ HRV^c^ only	0.53 (0.02)	0.48 (0.03)	0.34 (0.04)	0.73 (0.04)
+ PF^d^ only	0.50 (0.02)	0.45 (0.03)	0.33 (0.04)	0.80 (0.04)
Multimodal (all)	0.56 (0.02)	0.51 (0.03)	0.37 (0.05)	0.68 (0.04)

a MAE: mean absolute error. All metrics except MAE are higher-is-better.

b EEG: electroencephalography.

c HRV: heart rate variability.

d PF: psychological features.

### Predicted Alcohol Craving Values by Both Models

Figure 4 illustrates representative examples comparing predicted alcohol craving levels from the unimodal and multimodal models against the ground truth, respectively. For each prediction time point, the final predicted value was obtained by taking the mode of the 100 model outputs across different random seeds, with ties resolved by selecting the lower class label. Each row illustrates a distinct performance pattern. For participant 3, the multimodal model showed near-perfect tracking, whereas the unimodal model showed consistent deviation. For participant 6, the multimodal model achieved 5 of 5 correct predictions, compared with 2 of 5 for the unimodal model. For participant 27, both models showed comparable overall accuracy, but the multimodal model produced predictions closer to the true ordinal labels. For participant 44, high-craving minority class instances were better captured by the multimodal model. For participant 60, the multimodal model showed substantial accuracy improvement.

Overall, the multimodal model demonstrates more accurate and stable predictions compared to the unimodal model across participants. In several cases, the multimodal model achieves perfect or near-perfect accuracy, whereas the unimodal model exhibits substantial deviations from the true values. Importantly, the advantage of the multimodal model is not limited to overall accuracy. In cases where both models produce incorrect predictions, the multimodal model tends to generate predictions that are closer to the true ordinal label. This behavior is reflected in the lower MAE observed in the multimodal setting and suggests improved sensitivity to the ordinal structure of the target variable.

Furthermore, the multimodal model shows improved performance in capturing the minority class instances, allowing it to better capture less frequent but clinically important states. This is consistent with the observed improvement in the macro *F*_1_-score. Although some participants exhibit limited improvement in overall accuracy, the multimodal model still produces predictions that are closer to the ground truth, indicating meaningful improvements beyond simple accuracy gains. Taken together, these results suggest that incorporating static baseline features enables the model to generate more precise, balanced, and clinically informative predictions at the individual level.

**Figure 4.**
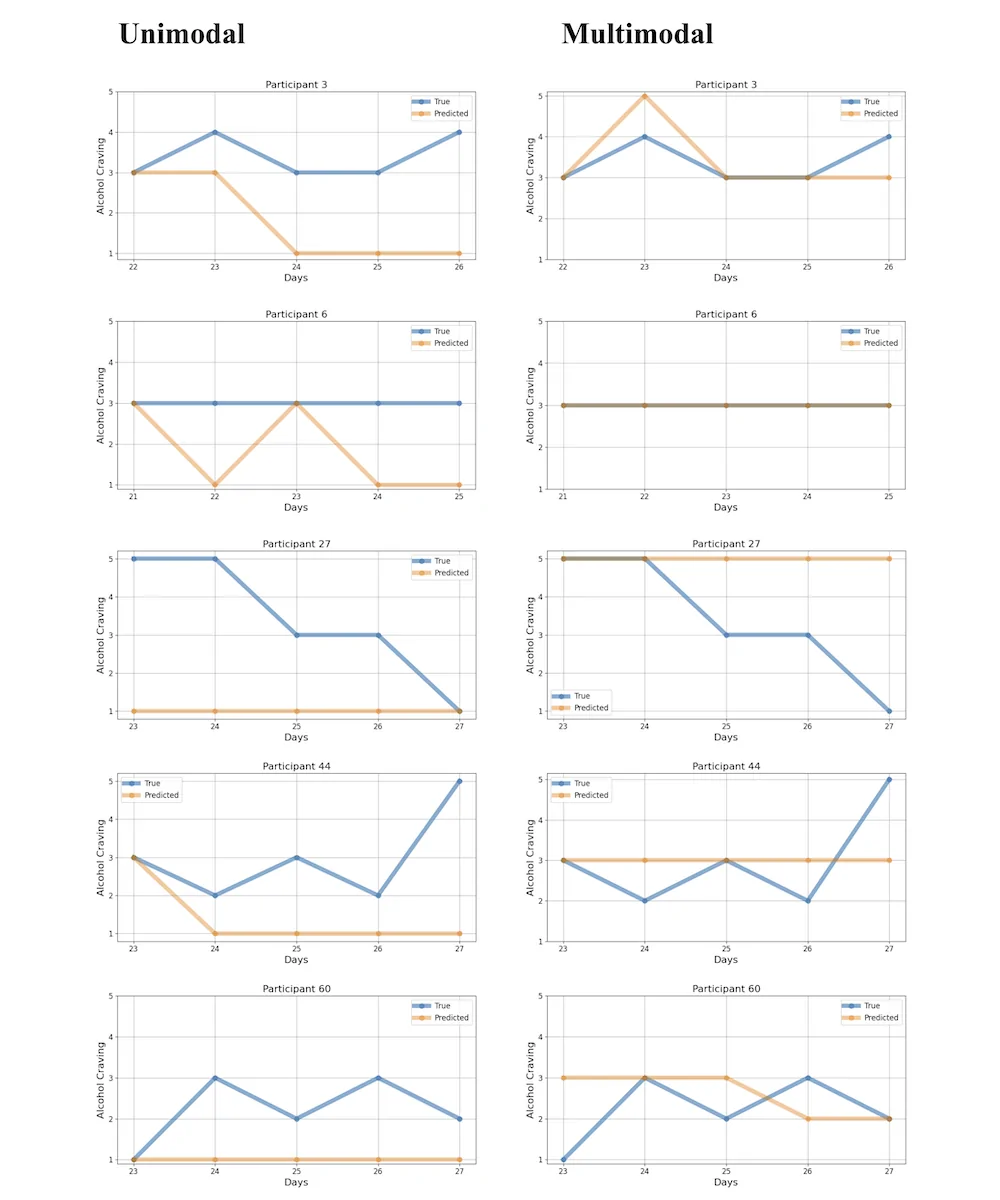
Representative participant-level comparisons of true and predicted next-day alcohol craving levels for the unimodal and multimodal models across five test days. Blue lines indicate true values, and orange lines indicate predicted values. The left column shows unimodal model predictions, and the right column shows multimodal model predictions. Final predictions were obtained by taking the mode across 100 random seed runs.

## Discussion

### Principal Findings

This study demonstrates that a multimodal AI approach improves the prediction of changes in next-day alcohol cravings over a 28-day period compared to a unimodal model based solely on digital phenotypes. By integrating digital behavioral data with neurophysiological and psychological features, the multimodal model achieved more accurate and consistent predictions across individuals. These findings suggest that static baseline characteristics provide subject-specific contextual information that shapes the interpretation of temporal behavioral patterns. By conditioning temporal predictions on individual-specific features, the model is better able to capture heterogeneity in craving dynamics across participants. Rather than assuming uniform temporal patterns across individuals, the multimodal approach enables more personalized and stable predictions of alcohol craving.

### Comparison to Prior Work

This study represents a novel attempt to integrate digital phenotypes collected through a smartphone app with various modalities using AI methods. A previous review study showed that smartphone-based digital phenotyping studies in AUD primarily use passive sensor data (eg, accelerometer, GPS, and app usage) to capture behavioral patterns such as movement and sociability, and rely mainly on self-reported alcohol use, craving, or mood [75]. Another study explored the association between smartphone-derived behavioral features and alcohol craving using EMAs [76]. That study demonstrated that sensor-derived features, such as location entropy and accelerometer-based activity, were significantly correlated with alcohol craving and mood states in individuals with AUD [76]. By contrast, this study advances this line of research by adopting a multimodal AI approach that enables individualized forecasting of daily alcohol craving. This study suggests that integrating all modalities provides better performance than using any single modality alone. This may be because the integration of these modalities captures diverse aspects of craving. This approach may not only improve predictive performance but also enhance the potential for clinical interventions.

While several approaches have been proposed to integrate static data with time-series data through feature concatenation [62,77], only a limited number of studies have explored incorporating static information by using feed-forward networks to summarize static features and initialize the hidden state of recurrent models [61,78]. While prior clinical studies adopting this framework predominantly rely on long short-term memory (LSTM) or gated recurrent unit (GRU)–based architectures, we demonstrate that a tiny lightweight vanilla RNN can achieve stable predictive performance in a small-sample multimodal setting. Given the short input sequence (window size=4) and limited sample size, we prioritized a lightweight vanilla RNN to minimize model complexity, consistent with the prior work noting that simpler RNN architectures require fewer computational resources and may be suitable for smaller datasets or short-term prediction settings [79]. LSTM architectures were originally developed to address difficulties in learning dependencies over extended temporal intervals [80], whereas gated recurrent architectures introduce additional parameters relative to vanilla RNNs [81]. Prior clinical time-series forecasting work has shown that vanilla RNN cells can remain competitive with LSTM and GRU cells while requiring fewer parameters and computational resources [81]. Accordingly, our primary aim was to evaluate multimodal integration within a deliberately compact recurrent framework rather than to identify the optimal sequence-learning architecture.

### Interpretation of the Findings

Combining all modalities yielded the greatest improvement in predictive performance compared with models based on each unimodality in predicting daily alcohol craving levels. In additional analyses, combining digital phenotype data with EEG or HRV improved model performance compared with using psychological features alone. A previous digital phenotype study showed that a combination of multimodal measurement and an AI approach has enhanced the performance of digital markers in phenotyping and diagnosis of major depressive disorder [82]. Prior work has similarly demonstrated that multimodal approaches outperform single-modality models in the diagnosis of internet gaming disorder and AUD, suggesting that integrating self-report–based neuropsychological measures with objective EEG-based neurophysiological data can enhance clinical assessment [83]. The improved performance of the multimodal AI model likely reflects the inherently multidimensional nature of alcohol craving, encompassing psychological, behavioral, and neurophysiological aspects [8,12,84]. Integrating frequency-specific EEG relative power features that reflect neurophysiological processes associated with craving, autonomic nervous system activity reflected by HRV, and subjective self-reports may provide complementary information that is critical for accurate prediction. Future research should continue to use multimodal frameworks to better account for the complex mechanisms underlying alcohol craving.

Post hoc analyses suggested that greater within-person craving variability and more diverse craving-level distributions were associated with lower participant-level prediction accuracy, whereas EMA completion and the number of usable sequential samples were not significantly associated with accuracy. Participants with greater craving variability and more diverse craving-level distributions may represent a more challenging forecasting problem. However, these associations were exploratory and should not be interpreted as causal.

Recently, just-in-time adaptive interventions have gained increasing attention as an approach to delivering interventions in real time by leveraging dynamically changing information about an individual’s state and context [85]. They adapt intervention strategies based on both dynamic data (eg, emotions, behaviors, and context) and stable characteristics (eg, demographics and baseline conditions) [85]. Our model may serve as a predictive component within the just-in-time adaptive interventions by identifying high-risk moments based on dynamic and individual-level data. For example, when predicted craving exceeds a predefined threshold, the system may trigger personalized interventions such as behavioral prompts or clinician alerts. Given that HRV alone provided substantial performance gains, a cost-effective deployment using wearable devices may be feasible without requiring full EEG acquisition. Although the sample size was relatively small, future studies with larger populations may help further validate our multimodal AI approaches, enhancing its potential for real-world clinical application. Moreover, the use of a tiny RNN to address temporal misalignment across modalities may represent a promising strategy for future studies.

The use of a tiny RNN architecture may have contributed to stable model performance by reducing the risk of overfitting in a relatively small sample size and short sequence settings. While more complex architectures such as LSTM or transformer models may offer additional flexibility, given the limited data and the use of short sliding windows, these models may introduce additional model complexity and a large number of parameters. Despite its simplicity, the present approach was sufficient to capture meaningful temporal dependencies in multimodal data.

### Limitations

Several limitations of this study should be noted. First, the sample size was relatively small to be considered representative of all problematic alcohol drinkers, which may limit the generalizability of the findings. Therefore, further investigations with larger sample sizes should be warranted. Second, this study was conducted in a single South Korean cohort without external validation using an independent dataset. This may limit the generalizability of the findings to other populations and settings. Future studies should therefore validate the model in independent and more diverse cohorts. Third, alternative sequence-learning and machine-learning approaches, including LSTM, GRU, temporal convolutional networks, and gradient-boosting methods, were not systematically evaluated in this study. We intentionally prioritized a lightweight vanilla RNN given the short temporal window and limited sample size; however, direct comparisons across alternative model classes are needed to determine whether the observed multimodal benefits generalize across architectures. Fourth, although only 29 (48.3%) of 60 participants provided EMA responses for exactly 28 days, all participants in the final analytic sample contributed at least 21 days of EMA data (range 21‐30 days). Within this observed range, EMA response days were not significantly associated with participant-level accuracy. Therefore, this study could not determine a lower minimum EMA completion threshold, such as 8 days, required for satisfactory prediction performance. Fifth, the overall predictive performance was modest, as reflected in the accuracy and weighted and macro *F*_1_-scores, indicating the need for further model optimization and validation. Sixth, next-day forecasting may not fully capture individual and circadian variability in craving dynamics; therefore, future studies should consider more frequent assessments and alternative forecasting intervals [16].

### Future Directions

Validation using larger and more diverse cohorts is necessary to enhance the generalizability of the model across broader populations of individuals with problematic alcohol use, particularly by incorporating heterogeneous clinical profiles and varying levels of craving variability. Second, future research should focus on improving model performance for individuals with high intra-individual variability in craving, potentially through advanced modeling approaches that better capture temporal dynamics, such as sophisticated sequence models or attention-based architectures.

Finally, integrating predicted high-risk moments of craving with timely and personalized interventions—such as alerts, behavioral strategies, or therapeutic support—may enable the model to serve as a core component of a digital therapeutic system for preventing alcohol use and relapse. Building on this study, future work will focus on developing an app-based intervention program for alcohol craving regulation that uses the predictive model to deliver real-time personalized support.

### Conclusion

This study attempts a novel approach to predicting daily alcohol craving in individuals with problematic alcohol use using AI algorithms that integrate digital phenotypes and neurophysiological and psychological data. The multimodal model outperformed single-modality approaches in predicting daily craving levels. Notably, integrating all modalities showed better performance than models combining digital phenotypes with either EEG, HRV, or psychological features. This study emphasizes the importance of multimodal approaches for daily alcohol craving prediction and suggests that these approaches may enable timely interventions to prevent problematic alcohol use.

## Supplementary material

10.2196/101973 Multimedia Appendix 1 Supplementary materials.

## Abbreviations

AUD: alcohol use disorder
AUDIT: Alcohol Use Disorders Identification Test
BAI: Beck Anxiety Inventory
BDI-II: Beck Depression Inventory-II
BIS: Barratt Impulsiveness Scale
DMQ: Drinking Motives Questionnaire
EEG: electroencephalography
EMA: ecological momentary assessment
GRU: gated recurrent unit
HRV: heart rate variability
LSTM: long short-term memory
MAE: mean absolute error
ML: machine learning
PSQI: Pittsburgh Sleep Quality Index
RNN: recurrent neural network
SOCRATES: Stages of Change Readiness and Treatment Eagerness Scale
