# Supplementary material for: Forecasting Next-Day Alcohol Craving in Individuals With Problematic Alcohol Use With Multimodal Data and a Tiny Recurrent Neural Network: Prospective Longitudinal Study
Source: J Med Internet Res. 2026 Nov 11;28:e101973. doi: 10.2196/101973 (PMC13754260; doi:10.2196/101973)
Supplement: Supplementary file 1 [file jmir-v28-e101973-s001.docx]

**Multimedia Appendix**

**Selected EMA and Baseline Features**

**Table S1**. Selected EMA features (11 total) collected daily via the Harujjan smartphone application over 28 days. Likert-scale items are ordinal; consumption and sleep duration are continuous.

| **Category** | **Feature** | **Question & Scale** |
| --- | --- | --- |
| *Mood (2 features)* | | |
| Mood | PHQ-1 | How did you feel today?  (1 = Very Sad -- 5 = Very Happy) |
| Mood | PHQ-2 | How much interest or pleasure did you experience today?  (1 = None -- 5 = Very Much) |
| *Diet (4 features)* | | |
| Diet | Meal_Morning | What is the quantity of your breakfast?  (0 = None, 1 = Little, 2 = Normal, 3 = Large) |
| Diet | Meal_Lunch | What is the quantity of your lunch?  (0 = None, 1 = Little, 2 = Normal, 3 = Large) |
| Diet | Meal_Dinner | What is the quantity of your dinner?  (0 = None, 1 = Little, 2 = Normal, 3 = Large) |
| Diet | Meal_Night | What is the quantity of your night snack?  (0 = None, 1 = Little, 2 = Normal, 3 = Large) |
| *Alcohol Consumption (2 features)* | | |
| Alcohol | Amount_Beer_Glass | Amount of beer consumed today (glasses) |
| Alcohol | Amount_Soju_Glass | Amount of soju consumed today (glasses) |
| *Sleep (3 features)* | | |
| Sleep | Sleep_Disturb | How much did last night's sleep interfere with your day today? (1 = None -- 5 = Very Much) |
| Sleep | Sleep_Satisfy | How satisfying was your sleep last night?  (1 = Very Bad -- 5 = Very Good) |
| Sleep | Sleep_Minute | How much did you sleep last night? (minutes) |

**Table S2**. Complete list of selected baseline features (41 total) used to initialize the RNN hidden state. Resting-state EEG features represent relative band power at individual electrodes. HRV features are derived from resting-state recordings. Psychological and cognitive features are from standardized assessments administered at baseline.

| **Category** | **Feature** | **Description** |
| --- | --- | --- |
| *Cognitive & Alcohol Use (6 features)* | | |
| Cognitive | VCI | Verbal Comprehension Index (K-WAIS-IV) |
| Cognitive | PRI | Perceptual Reasoning Index (K-WAIS-IV) |
| Cognitive | WMI | Working Memory Index (K-WAIS-IV) |
| Cognitive | IQ | Full-Scale Intelligence Quotient (K-WAIS-IV) |
| Alcohol use | alcohol_freq | Alcohol use frequency |
| Alcohol use | pure_alcohol | Mean pure alcohol intake (g/day) |
| **Psychological Scales (5 features)** | | |
| DMQ | DMQ_Social | Drinking Motives Questionnaire — Social motives |
| DMQ | DMQ_Coping | Drinking Motives Questionnaire — Coping motives |
| DMQ | DMQ_Conformity | Drinking Motives Questionnaire — Conformity motives |
| BIS | BIS_Total | Barratt Impulsiveness Scale — Total score |
| SOCRATES | SOCRATES_Amb | SOCRATES — Ambivalence subscale |
| **EEG — Relative Band Power (22 features)** | | |
| Delta (1–4 Hz) | delta-T7, delta-CP6, delta-P2 | Relative delta power at T7, CP6, P2 |
| Theta (4–8 Hz) | theta-C4, theta-CPz, theta-TP8, theta-Oz | Relative theta power at C4, CPz, TP8, Oz |
| Alpha (8–12 Hz) | alpha-Fp1, alpha-F7 | Relative alpha power at Fp1, F7 |
| Beta (12–25 Hz) | beta-F5, beta-F1, beta-F6, beta-FCz, beta-T8, beta-CP5, beta-Oz | Relative beta power at F5, F1, F6, FCz, T8, CP5, Oz |
| Gamma (30–40 Hz) | gamma-F3, gamma-FC2, gamma-T7, gamma-TP7, gamma-P7, gamma-O2 | Relative gamma power at F3, FC2, T7, TP7, P7, O2 |
| **HRV — Heart Rate Variability (8 features)** | | |
| HRV | ANS_activity | Autonomic nervous system activity index |
| HRV | ANS_balance | Autonomic nervous system balance index |
| HRV | cardiac_stability | Cardiac stability index |
| HRV | Tp | Total power |
| HRV | ApEn | Approximate entropy |
| HRV | Srd | Short-range detrended fluctuation analysis |
| HRV | Vlf(ln) | Natural log of very low-frequency power |
| HRV | cardiac_state | Cardiac stability state (encoded categorical) |

**Training Dynamics**

## **Figure S1.** Training vs validation loss curves averaged over 100 runs (shaded region: ±1 SD). The dashed vertical line indicates the median best epoch selected by early stopping. Left: Unimodal model (median best epoch = 17, IQR: 13-21), which reaches its optimum early and subsequently shows increasing validation loss, indicating earlier onset of overfitting. Right: Multimodal model (median best epoch = 28; IQR: 19-36), which converges more slowly but maintains stable validation loss after the optimal epoch, reflecting more robust generalization

|  |  |
| --- | --- |
| The multimodal model’s slower convergence is attributable to the increased model complexity introduced by incorporating static baseline features. Importantly, this translates into more stable learning behavior rather than overfitting, despite the limited sample size (N = 60). | |

**Figure S2.** Training vs validation accuracy curves averaged over 100 runs (shaded region: ±1 SD). The dashed vertical line indicates the median best epoch selected by early stopping. Left: Unimodal model (shaded region: ±1 SD) – validation accuracy peaks near epoch 17 then declines, while training accuracy continues to rise, indicating overfitting. Right: Multimodal model – validation accuracy remains relatively stable after the optimal epoch (median = 28), suggesting that baseline-informed hidden state initialization acts as an inductive bias that improves generalization.

|  |  |
| --- | --- |
| Taken together, these training dynamics suggest that although the multimodal model requires more epochs to converge, it is less prone to rapid overfitting – a meaningful advantage given the small sample size of this study. | |

**Distribution of EMA Response Imbalance**

## **Figure S3. Distribution of EMA response days and craving label imbalance.**

## Left: Number of days with EMA responses across participants in the final analytic sample (N = 60). Twenty-nine participants (29 of 60) provided EMA responses for exactly 28 days, and the number of EMA response days ranged from 21 to 30 across participants. Right: Distribution of alcohol craving levels (1-5 Likert scale) in the lagged dataset used to construct next-day prediction samples. The distribution is markedly skewed toward lower craving levels, highlighting substantial class imbalance that motivated the use of macro F1-score and MAE as complementary evaluation metrics alongside accuracy.

|  |  |
| --- | --- |

**Participant-Level Factors Associated with Prediction Accuracy**

## **Table S3. Associations between participant-level EMA characteristics and prediction accuracy.**

| **Participant  characteristics** | **Unimodal accuracy ρ** | **P value** | **Multimodal accuracy ρ** | **P value** | **ΔAccuracy ρ** | **P value** |
| --- | --- | --- | --- | --- | --- | --- |
| EMA response days | 0.035 | .788 | 0.085 | .519 | 0.041 | .756 |
| Craving SD | -0.549 | <.001 | -0.671 | <.001 | 0.008 | .949 |
| Craving entropy | -0.533 | <.001 | -0.688 | <.001 | -0.040 | .763 |
| Number of unique craving levels | -0.445 | <.001 | -0.535 | <.001 | -0.008 | .953 |
| Valid training samples | 0.087 | .510 | 0.107 | .415 | 0.070 | .596 |
| Total valid samples | 0.087 | .510 | 0.107 | .415 | 0.070 | .596 |

Values are Spearman rank correlation coefficients (ρ). Participant-level accuracy represents mean test-set accuracy across 100 model runs with different random seeds. ΔAccuracy was calculated as multimodal accuracy minus unimodal accuracy. Craving SD represents within-participant variability in craving ratings, whereas craving entropy reflects the diversity and evenness of the within-participant craving-level distribution. Valid training samples and total valid samples represent usable sequential samples remaining after sliding-window construction and temporal-validity filtering. All participants in the final analytic sample contributed 21-30 days of EMA data.

Valid test-sample counts showed minimal between-participant variation, with 59 of 60 participants contributing 5 valid test samples and 1 participant contributing 4.

**Classification Performance by Craving Level**

## **Figure S4. Confusion matrices for next-day alcohol craving prediction in the test set**

Confusion matrices show the observed and predicted alcohol craving levels (1-5) across the full test set for the unimodal and multimodal models. For each test sample, the predicted craving level was defined as the modal prediction across 100 model runs with different random seeds, consistent with the procedure used for the individual-level visualization in Figure 4. This differs from the aggregate performance metrics reported in Table 2, which are summarized across the 100 runs using the mean, SD, and 95% CI of the mean. Rows represent the true observed craving levels, and columns represent the predicted craving levels. (A) Raw-count confusion matrix for the unimodal model. (B) Raw-count confusion matrix for the multimodal model. (C) Row-normalized confusion matrix for the unimodal model. (D) Row-normalized confusion matrix for the multimodal model. In the row-normalized matrices, each cell represents the proportion of samples within a given observed craving level that were assigned to each predicted craving level.

|  |  |
| --- | --- |
| A. **Unimodal model – (Raw counts)** | B. **Multimodal model – (Raw counts)** |
|  |  |
| C. **Unimodal model – (Row-normalized)** | D. **Multimodal model – (Row-normalized)** |

**Window-Size Sensitivity Analysis**

We conducted a sensitivity analysis comparing sliding window sizes of 2, 4, 6, and 8 days using the validation set.

## **Table S4. Performance across sliding window sizes on the validation set**

|  | **Unimodal** | | | | **Multimodal** | | | | | |
| --- | --- | --- | --- | --- | --- | --- | --- | --- | --- | --- |
| **Window** | Acc | Weighted-F1 | Macro-F1 | MAE | | Acc | Weighted-F1 | | Macro-F1 | MAE |
| 2 | 0.47 | 0.39 | 0.22 | 0.83 | 0.53 | | 0.51 | 0.36 | | 0.71 |
| 4 | 0.46 | 0.39 | 0.23 | 0.85 | 0.53 | | 0.49 | 0.32 | | 0.72 |
| 6 | 0.46 | 0.38 | 0.22 | 0.88 | 0.52 | | 0.46 | 0.29 | | 0.74 |
| 8 | 0.46 | 0.38 | 0.22 | 0.89 | 0.50 | | 0.43 | 0.26 | | 0.80 |
| Note. Values represent means across 100 runs. Acc: accuracy; MAE: mean absolute error. All metrics except MAE are higher-is-better. | | | | | | | | | | |

The 2-day window yielded numerically higher performance on several validation metrics. However, differences between the 2- and 4-day windows were generally modest: multimodal accuracy was identical (0.53 for both), unimodal weighted F1-score was also identical (0.39 for both), and the 4-day window yielded slightly higher unimodal macro F1-score (0.23 vs 0.22). In contrast, performance generally declined with longer windows of 6 and 8 days, particularly in the multimodal model. These findings indicate that performance was relatively stable across short lookback windows, whereas longer windows tended to reduce performance.
